# Field Application of Layered Double Hydroxides to Reduce Cd Bioavailability and Uptake in *Artemisia argyi* Grown in Severely Contaminated Soil

**DOI:** 10.3390/toxics14060476

**Published:** 2026-05-29

**Authors:** Wei Qiu, Yujuan Huang, Chen Tu, Shuai Yang, Yi Wang, Xia Zhu, Yongming Luo

**Affiliations:** 1 State Key Laboratory of Soil and Sustainable Agriculture, Institute of Soil Science, Chinese Academy of Sciences, Nanjing 211135, China; qiuwei@issas.ac.cn (W.Q.); yjhuang@issas.ac.cn (Y.H.); ctu@issas.ac.cn (C.T.); syang@issas.ac.cn (S.Y.); yiwang1208@163.com (Y.W.); zhuxia@issas.ac.cn (X.Z.); 2 University of Chinese Academy of Sciences, Beijing 100049, China

**Keywords:** *Artemisia argyi*, layered double hydroxides (LDHs), non-food cropping, severely Cd-contaminated farmland, moxa smoke

## Abstract

Non-food cropping provides a practical strategy for the safe utilization of severely cadmium (Cd)-contaminated farmland. In this study, a field experiment was conducted to evaluate the effectiveness of layered double hydroxides (LDHs) in reducing Cd transfer from soil to *Artemisia argyi*, a plant used for non-food applications, and to estimate Cd release potential during moxa burning. Our results demonstrated that the application of LDHs increased soil pH and decreased the extractable Cd concentration based on CaCl_2_ extraction, suggesting a reduction in Cd bioavailability. Furthermore, BCR fractionation analysis indicated a shift of Cd from more active to more stable forms, further supporting the reduction in Cd bioavailability in the soil. SEM–EDS and FTIR confirmed the lamellar morphology, CaAl composition, and hydroxyl-rich functional groups of the LDH conditioner. Plant growth was not negatively affected by LDH treatment, and Cd concentrations in roots, stems, and leaves were significantly reduced. LDHs also reduced Cd levels in processed moxa and the mass-balance-based estimate of Cd release during combustion. These findings suggest that LDHs application may help reduce Cd transfer in non-food cropping systems on severely contaminated farmland.

## 1. Introduction

Soil contamination by heavy metals is a widespread environmental and public health issue, threatening ecosystem stability and the sustainability of agricultural production [1,2]. Cadmium (Cd), in particular, is a major concern in China’s farmland soils due to its high mobility and persistence, which makes it readily absorbed by crops and transferred through the food chain. Continued Cd accumulation in soil exacerbates its movement into the food chain, increasing both ecological and human health risks [3,4,5]. To mitigate these risks, China has developed a risk-based framework for the classification and management of farmland, with GB 15618 (2018) serving as the regulatory basis [6]. This framework classifies heavily contaminated farmland as “strictly controlled”, where the cultivation of edible crops is prohibited [7]. However, the restoration of edible crop production in these areas is often challenging. By contrast, non-food cropping systems offer a feasible alternative, enabling the continued use of contaminated farmland while minimizing dietary exposure [8,9,10].

*Artemisia argyi*, a perennial medicinal plant [11,12,13], has garnered increasing attention for its adaptability to various environmental conditions, low soil requirements, and tolerance to heavy metal contamination [14,15]. Previous studies have shown that *A. argyi* can maintain high biomass and stable physiological performance under Cd stress, with the ability to accumulate Cd in its tissues [16,17]. Importantly, the aboveground parts of *A. argyi* are primarily used to produce non-food products, such as moxa for moxibustion and essential oils, which do not enter the food chain [18]. This makes it a suitable crop for risk-oriented utilization in heavily contaminated farmland, where non-food crops are preferred. However, unlike food crops, *A. argyi* is processed and then used in combustion, raising the concern that Cd could be released into smoke during burning [19,20]. The resulting smoke contains particulate matter that may carry metal contaminants, posing potential health risks [21,22,23,24]. Given that *A. argyi* can accumulate Cd, it is crucial to understand whether Cd is released during combustion.

To address this, stabilization remediation techniques are used to reduce the bioavailability of Cd in contaminated soils [25,26,27]. Among various soil conditioners, layered double hydroxides (LDHs) have shown promise due to their ability to stabilize cationic heavy metals like Cd through adsorption and ion exchange mechanisms [28,29,30,31]. Previous studies have primarily focused on soils with low to moderate Cd contamination, with most research examining Cd concentrations in grain crops and vegetables [32,33,34,35,36]. However, limited evidence exists regarding the effectiveness of LDHs in reducing Cd accumulation in *A. argyi* grown on heavily contaminated soils, and whether this results in lower Cd concentrations in processed products or reduced Cd release during combustion.

**Figure 1:**
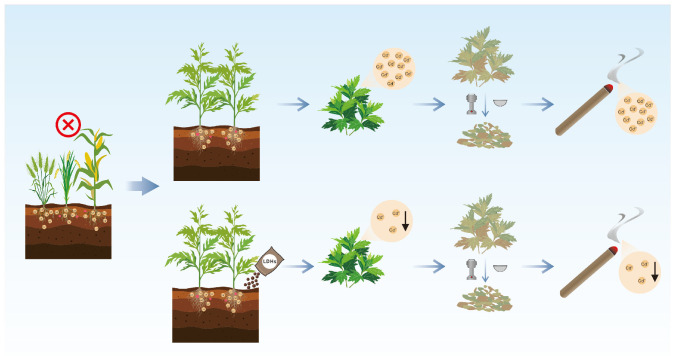
Conceptual diagram of LDH-mediated risk control during *A. argyi* cultivation, processing, and utilization in Cd contaminated soil. The red ⊗ indicates that edible crop cultivation is restricted on severely Cd-contaminated farmland under strict risk control.

This study aims to fill this gap by investigating the application of LDHs in a field experiment on severely Cd-contaminated farmland using *A. argyi* as a substitution crop. We evaluate the effect of LDHs on Cd distribution in soil and plant tissues, as well as its impacts on Cd levels in processed moxa and estimated Cd release potential during combustion. Soil pH, Cd bioavailability based on CaCl_2_ extraction and the European Community Bureau of Reference (BCR) fractionation, and Cd concentrations in plant tissues and processed moxa were measured. Finally, Cd release during moxa combustion was estimated using a mass-balance approach (Figure 1), rather than being interpreted as a full inhalation exposure or health risk assessment. This study provides valuable insights into the management of severely contaminated farmland through non-food cropping systems and the safe use of contaminated land.

## 2. Materials and Methods

### 2.1. Site Characterization

**Figure 2:**
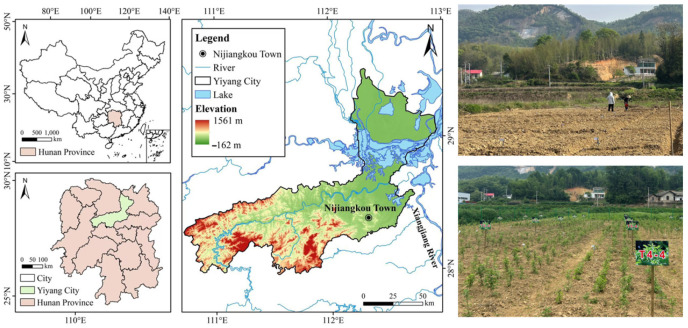
Schematic diagram of the trial area in Yiyang city, Hunan province, China.

The study was conducted in Nijiangkou Town, Yiyang City, Hunan Province, China (Figure 2). This region is characterized by a subtropical monsoon humid climate, with a mean annual sunshine duration of 1550.1 h. The mean annual temperature is 19.1 °C, and the mean annual precipitation is 1161.0 mm. The soil at the site is classified as paddy soil. This area has a long history of mining and smelting related to non-ferrous metal production, which has led to the release of large amounts of Cd and other heavy metals into the environment, with atmospheric deposition considered a major pathway. Over time, Cd has accumulated in the surface soils. The initial soil properties of the tilled layer (0–20 cm) were as follows: soil organic matter (SOM) 26.7 g kg^−1^, total nitrogen (TN) 1.86 g kg^−1^, total phosphorus (TP) 0.35 g kg^−1^, total potassium (TK) 18.1 g kg^−1^, alkali-hydrolyzed nitrogen (AN) 0.19 g kg^−1^, available phosphorus (AP) 0.03 g kg^−1^, available potassium (AK) 0.16 g kg^−1^, pH was 4.67, and the total Cd concentration 7.04 mg kg^−1^. According to the soil contamination risk control standard of agricultural land (GB 15618-2018), the soil is classified as severely contaminated with Cd [6,7].

### 2.2. Materials and Field Experimental Design

*A. argyi* seedlings used in this study were obtained from a local agricultural cooperative. All seedlings exhibited uniform growth and showed no visible signs of pests or diseases. The CaAl-LDH conditioner, designated as LDHs, was supplied by Jiangsu Longchang Chemical Co., Ltd., Rugao City, China, and used as the soil conditioner in this study. A control without soil conditioner was also included and designated as CK. The soil conditioner had a pH of 11.89 and a total Cd concentration of 0.08 mg kg^−1^, and it was applied at a rate of 4500 kg ha^−1^. The CaAl-LDH conditioner was characterized before field application by scanning electron microscopy coupled with energy-dispersive spectroscopy (SEM–EDS, EVO 18, Zeiss, Jena, Germany) and Fourier-transform infrared spectroscopy (FTIR, Nicolet iS10, Thermo Fisher, Waltham, MA, USA). SEM–EDS was used to examine the surface morphology, layered structure, and elemental composition of the conditioner, while FTIR was used to identify the main functional groups. A randomized complete block design was used for the field experiment. The two treatments, LDHs and CK, were randomly arranged within each block, with three replicates per treatment. Each plot covered an area of 16.0 m^2^ (4.0 m × 4.0 m). Trenches were established between plots to minimize water and nutrient movement across treatments, and buffer rows were placed around the experimental area to reduce edge effects.

The conditioner was uniformly applied to the soil surface in the LDH treatment plots on 9 April 2025 and incorporated into the topsoil to a depth of approximately 20 cm. *A. argyi* seedlings were transplanted on 17 April 2025 at predefined row and plant spacing, with 88 plants per plot. Plant samples were collected at harvest on 28 July 2025 for subsequent analyses. The cropping period from transplantation to harvest lasted approximately 102 days, while the period from LDH application to harvest lasted approximately 110 days. Throughout the growing period, irrigation and weed control were performed as needed.

### 2.3. Sampling and Measurement

Soil samples were collected from the 0–20 cm soil layer during the experiment. Within each plot, surface soil samples were collected from multiple randomly selected points and then composited into one representative sample to reduce the influence of within-plot heterogeneity. After collection, the samples were air-dried, plant residues removed, and sieved through 2 mm and 0.15 mm mesh nylon sieves for the analysis of physicochemical properties and Cd contents. Soil physicochemical properties were analyzed following the method described by Bao [37]. Available Cd in soil was extracted using 0.01 mol L^−1^ CaCl_2_ at a ratio of 1:10 (*w*:*v*) [38,39]. Soil Cd fractions were determined using the modified European Community Bureau of Reference (BCR) sequential extraction method [40]. Briefly, the exchangeable fraction (EX-Cd) was extracted with 0.11 mol L^−1^ acetic acid, representing the readily soluble and weakly bound Cd fraction. The reducible fraction (RE-Cd) was extracted with 0.5 mol L^−1^ hydroxylamine hydrochloride, mainly representing Cd associated with Fe/Mn oxides. The oxidizable fraction (OX-Cd) was extracted after oxidation with H_2_O_2_, followed by extraction with 1.0 mol L^−1^ ammonium acetate, representing Cd bound to organic matter and sulfides. The residual fraction (RS-Cd) was determined after strong acid digestion of the remaining residue, representing Cd incorporated into mineral lattices or other stable residual forms. After each extraction step, the suspension was centrifuged, and the supernatant was collected for Cd analysis by ICP-MS.

Plant samples were collected using a composite sampling method. At harvest, five *A. argyi* plants were randomly selected from each plot, and plant height was measured. The plants were then separated into leaves, stems, and roots, and fresh weights were recorded. The samples were washed with tap water followed by deionized water, air dried to remove surface moisture, and placed in kraft paper envelopes. After drying at 105 °C for 30 min, they were further dried at 75 °C to a constant weight for biomass measurement. Once dried, the samples were ground and passed through a 0.15 mm nylon mesh sieve for acid digestion and Cd analysis [41]. After removing the petioles and midribs, the leaves were ground with a high-speed grinder at 28,000 rpm for 30 s [42]. The ground material was then sieved through a 2 mm mesh, and the remaining fraction on the sieve was collected as the moxa sample.

Before Cd analysis, both soil and plant samples were digested using a graphite digestion system. Soil samples were digested with a mixture of HNO_3_, HClO_4_, and HF (2:1:1, *v*/*v*/*v*), and plant samples were digested with a mixture of HNO_3_ and HClO_4_, (10:1, *v*/*v*) [43]. Cd concentrations were determined by ICP-MS (NexION 2000, PerkinElmer, Waltham, MA, USA) in kinetic energy discrimination (KED) mode, with Rh (10 μg L^−1^) as the internal standard. Reagent blanks, duplicate samples, and certified reference materials were included in each digestion and analytical batch. The correlation coefficient of the Cd calibration curve was higher than 0.999. The method detection limit for Cd was 0.05 μg L^−1^. The recoveries of Cd in the certified reference materials GSS-72 and GBW10015a were 90–110% and 95–105%, respectively. For duplicate samples, the absolute difference between two measurements did not exceed 10% of their arithmetic mean.

### 2.4. Mass-Balance Estimation of Cd Release During Moxa Combustion

This study did not conduct a full quantitative inhalation exposure or health risk assessment during moxa combustion. Instead, Cd release during moxa burning was evaluated using a mass-balance-based release potential. In the experiment, a fixed mass of moxa was burned, and the residual ash was collected after combustion. Cd concentrations in moxa before burning and in residual ash after burning were determined. The estimated smoke-phase Cd was calculated as the difference between Cd in moxa before combustion and Cd retained in ash after combustion. The Cd release factor was expressed per unit mass of burned moxa and used to compare the relative difference in Cd release potential between the CK and LDH treatments.

### 2.5. Statistical Analysis

Data processing was performed using Microsoft Excel 2021. Results are presented as the mean ± standard deviation of three biological replicates. Statistical analyses were conducted using SPSS 2021. Before ANOVA, data normality and homogeneity of variance were tested using the Shapiro–Wilk test and Levene’s test, respectively. Treatment differences were assessed using one-way ANOVA at *p* < 0.05. Figures were generated using Origin 2024.

## 3. Results and Discussion

### 3.1. Characterization of the LDHs

**Figure 3:**
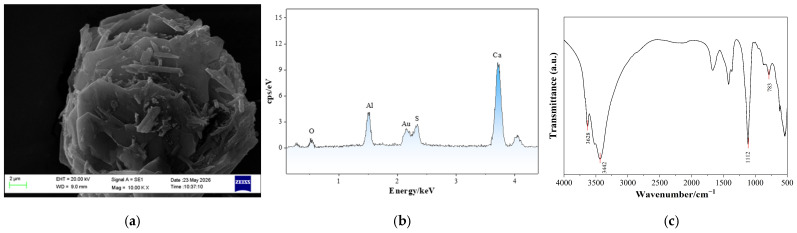
Characterization of the Ca–Al LDH conditioner. (**a**) SEM image, (**b**) EDS spectrum and elemental composition, and (**c**) FTIR spectrum. The Au signal in (**b**) was caused by sputter coating before SEM observation and was excluded from the elemental interpretation.

The sulfate-intercalated CaAl-LDH conditioner was characterized before field application (Figure 3). The SEM image showed aggregated plate-like particles with rough surfaces, displaying a layered morphology typical of LDH-type materials. These stacked and irregular lamellar structures indicated that the conditioner retained the basic layered characteristics of LDHs [36]. EDS analysis confirmed that Ca, O, Al, and S were the main detected elements in the conditioner, consistent with the Ca and Al composition and the presence of sulfate containing groups. The FTIR spectrum showed absorption bands at approximately 3628 and 3442 cm^−1^, which were assigned to O-H stretching vibrations of structural hydroxyl groups, surface hydroxyl groups, and interlayer water. The band near 1112 cm^−1^ was attributed to S-O stretching vibration of interlayer sulfate groups, while the band around 783 cm^−1^ was associated with metal–oxygen (M-O, mainly Ca-O and Al-O) lattice vibrations [44]. These results support the presence of lamellar Ca–Al-containing particles with hydroxyl- and sulfate-related functional groups.

### 3.2. Plant Biomass Under Cd Pollution

Soil conditioners are widely used in agriculture, but their effects on plant biomass and crop yield vary across studies [45]. Some research suggests that soil conditioners can promote biomass accumulation, while others report reductions in growth [46]. These variable effects have primarily been observed in vegetables [47] and major staple crops, such as rice [48], maize, and wheat [49]. However, *A. argyi*, a non-food medicinal plant, was investigated in this study. The results showed no significant differences in plant height or dry biomass of roots, stems, and leaves between the LDH treatment and the control (Table 1).

These results indicate that the soil conditioner did not significantly influence plant growth. This can be attributed to the fact that the LDHs used was developed primarily for Cd stabilization rather than nutrient provision. Consequently, the conditioner was unlikely to supply nutrients that would stimulate biomass accumulation [50,51].

### 3.3. Effects of LDHs on Soil pH, Cd Bioavailability, and Speciation

**Figure 4:**
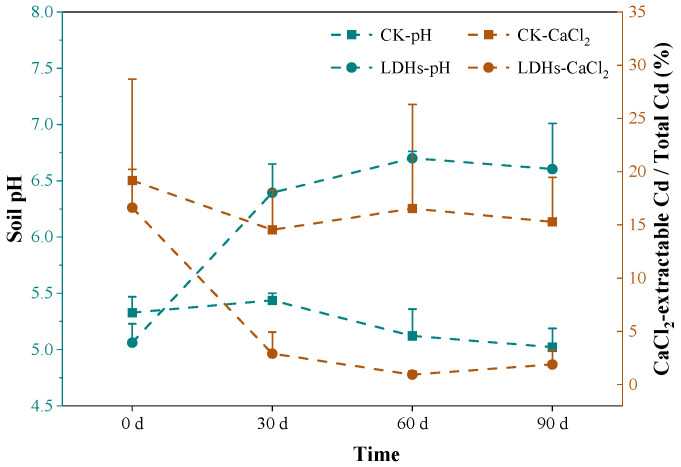
Temporal changes in soil pH and the proportion of CaCl_2_-extractable Cd in total soil Cd after LDH application.

Soil pH plays an important role in controlling Cd solubility, mobility, and chemical speciation [52]. As shown in Figure 4, the pH of both CK and LDH-treated soils was similarly acidic at the beginning of the experiment. After LDH application, soil pH increased significantly by 1.3 units and then remained relatively stable during the growing period. Meanwhile, CaCl_2_-extractable Cd decreased markedly after LDH application. Compared with the initial stage, CaCl_2_-extractable Cd decreased by 85%, with the most pronounced decline occurring at the early stage of the experiment. After this rapid decrease, CaCl_2_-extractable Cd gradually stabilized, suggesting that soil Cd availability reached a new equilibrium.

**Figure 5:**
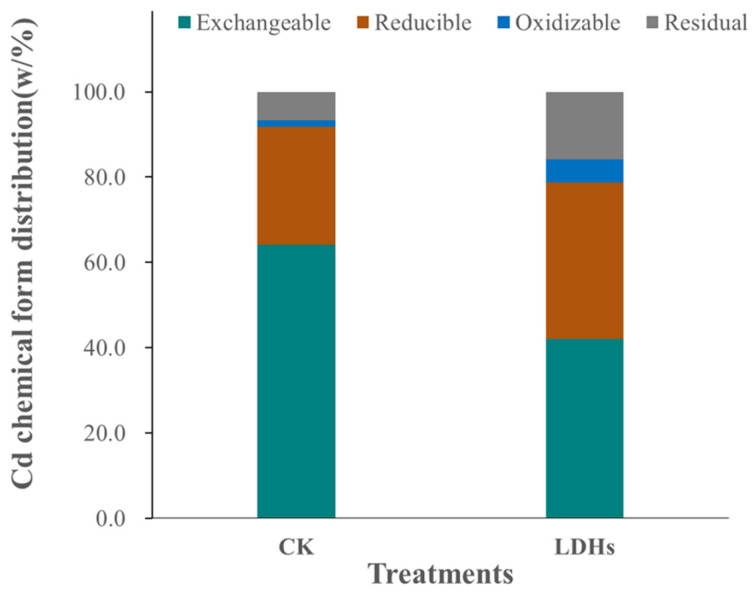
Distribution of Cd fractions in soil at harvest after LDH application.

The BCR fractionation results (Figure 5) further showed that LDHs changed the distribution of Cd fractions in soil. In the control soil, Cd was mainly present in the exchangeable fraction, accounting for 64.1% of total Cd, followed by the reducible fraction at 27.6%. The oxidizable and residual fractions accounted for 1.6% and 6.7%, respectively. After LDH application, the exchangeable Cd fraction decreased to 42.1%, while the reducible, oxidizable, and residual fractions increased to 36.6%, 5.4%, and 15.7%, respectively. These changes indicate that LDHs reduced the proportion of readily available Cd and promoted Cd redistribution toward relatively stable fractions.

The reduction in Cd bioavailability was likely associated with the increase in soil pH and the Cd retention capacity of the LDH material. Higher soil pH can decrease Cd^2+^ activity in soil solution and promote Cd retention in less soluble or less labile forms [53,54,55,56]. In addition, SEM–EDS and FTIR characterization showed that the LDH conditioner had a plate like morphology, Ca, Al and S elemental composition, and hydroxyl and sulfate related functional groups. These properties, together with the alkaline nature of the conditioner, may provide active sites for Cd retention [57,58,59,60,61]. However, because adsorption, ion exchange, coprecipitation, and structural incorporation were not directly tested in this study, these mechanisms should be regarded as possible explanations rather than directly demonstrated processes. Further studies combining XRD, BET surface area analysis, zeta potential measurements, particle size analysis, and Cd adsorption/desorption experiments are needed to verify the structural properties and Cd retention mechanisms of the LDH conditioner.

Lime can rapidly increase soil pH and reduce Cd mobility, but excessive or long-term application may increase the risk of soil hardening and biochar can immobilize Cd through adsorption and complexation, but relatively high application rates are often required in field practice [26]. In contrast, the CaAl-LDH conditioner used in this study was applied at a relatively low rate and combined alkalinity-related pH regulation with hydroxyl- and sulfate-related functional groups, which may contribute to Cd retention. Therefore, LDHs may have potential for Cd risk control in contaminated farmland, although further long-term comparative field studies are still needed.

### 3.4. Cd Accumulation and Translocation in Artemisia argyi

**Figure 6:**
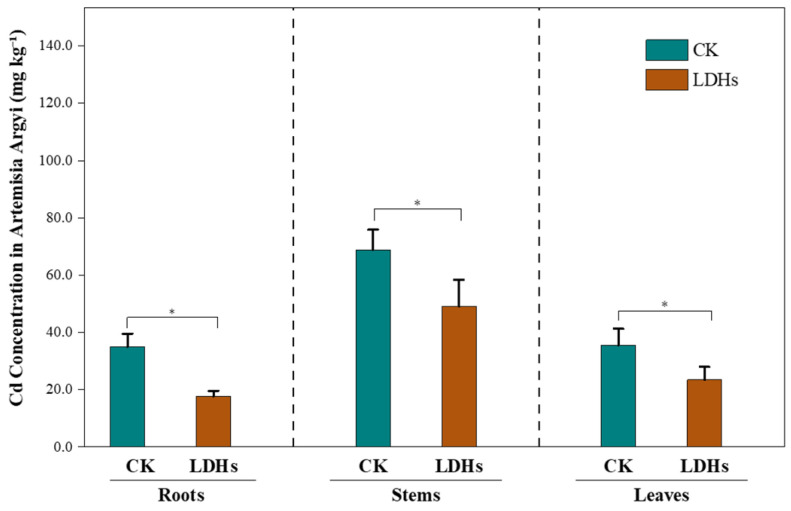
Cd concentrations in roots, stems, and leaves of *A. argyi* under CK and LDH treatments. The * indicates a significant difference between CK and LDHs treatments at *p* < 0.05.

The concentrations of Cd in the roots, stems, and leaves of *A. argyi* were measured to assess Cd accumulation and translocation. As shown in Figure 6, Cd concentrations were significantly higher in stems than in roots and leaves under both CK and LDH treatment. Compared with CK, LDH treatment resulted in a significant reduction in Cd concentrations across all plant tissues, with reductions of 49.1% in roots, 28.7% in stems, and 34.0% in leaves. This indicates that LDHs reduced Cd accumulation throughout the plant. The BCF further supported this finding, with lower BCF values in all plant tissues under LDH treatment, suggesting reduced Cd uptake and movement.

These results are consistent with previous studies, which have shown that lower soil Cd availability typically leads to reduced Cd concentrations in plant tissues [62,63]. In this study, LDHs reduced Cd availability in soil, which likely limited its uptake by roots and translocation to aboveground tissues. This pattern was supported by the soil indicators, as CaCl_2_ extractable Cd stayed at a lower level during the growing period. Cd also shifted from more labile forms to more stable ones, which further indicates that less Cd was available for plant uptake. Because root uptake is the main route by which Cd enters plants, the lower root bioconcentration under LDHs suggests that Cd transfer from soil to roots was reduced [64,65]. This may also be related to changes in rhizosphere ionic conditions. EDS analysis confirmed the presence of Ca in the LDH conditioner, suggesting that Ca related ionic competition may have contributed to limiting Cd^2+^ entry through shared or non-specific transport pathways [66].

### 3.5. Cd in Moxa and Estimated Cd Release During Combustion

In this study, LDH treatment reduced the Cd content in moxa by 32.0% compared with the control, indicating that the lower Cd accumulation in leaves was reflected in the processed moxa product. LDH treatment also reduced the estimated Cd release potential during combustion. As shown in Table 2, the mass-balance approach estimated that the Cd release factor during burning was 33% lower under the LDH treatment than under CK. These results indicate that reducing Cd accumulation during cultivation can lead to lower Cd content in processed moxa and a lower mass-balance-based estimate of Cd release during combustion.

These findings are consistent with the trends observed in soil and plant Cd dynamics. Following LDH application, soil Cd bioavailability declined, as indicated by lower CaCl_2_-extractable Cd and BCR fractionation results, which showed a redistribution of Cd from more labile to more stable fractions. Correspondingly, Cd concentrations in plant tissues decreased, and the processed moxa exhibited lower Cd content. During combustion, less Cd was estimated to enter smoke, which is relevant for product use safety because moxa smoke may act as a potential exposure medium under indoor conditions.

Inhalation of smoke borne Cd can pose health risks, especially in poorly ventilated indoor environments, such as those where moxibustion is typically performed. The mass-balance results provide a relative comparison of Cd release potential between the CK and LDH treatments. However, this approach cannot describe the actual airborne Cd concentration or exposure level during moxibustion. Smoke particles were not collected, and particulate matter concentration, aerosol size distribution, inhalation rate, exposure duration, and ventilation conditions were not measured. Therefore, the combustion-related results should be interpreted as an estimate of Cd release potential rather than a quantitative inhalation exposure or health risk assessment. Future studies should directly characterize Cd in smoke particles and combine air concentration measurements with exposure models to evaluate actual inhalation risks under typical moxibustion conditions.

## 4. Conclusions

This study investigated the role of LDHs in reducing Cd transfer from soil to *A. argyi,* processed moxa, and estimated Cd release during combustion on severely Cd-contaminated farmland. While LDHs did not significantly affect plant growth, as no notable changes were observed in plant height or biomass, they effectively altered Cd behavior in the soil. Specifically, LDHs increased soil pH and reduced CaCl_2_-extractable Cd, indicating a decrease in the bioavailability of Cd in the soil. Furthermore, Cd shifted from more active to more stable forms, enhancing Cd stabilization in the soil. The changes in soil Cd availability were reflected in plant Cd accumulation. Cd concentrations in roots, stems, and leaves were reduced under LDH treatment, and the Cd concentration in processed moxa was also reduced. In addition, the mass-balance-based estimate showed that less Cd entered the smoke during moxa combustion under LDH treatment. These findings should be interpreted with several limitations in mind. This study was conducted at a single field site, with one LDH application rate and one growing season. Therefore, the generality and reproducibility of the results should be further evaluated using multi-site and multi-year field experiments with greater replication.

## Figures and Tables

**Table 1:** *A. argyi* height and biomass of roots, stems, and leaves.

Treatments	Height (cm)	Dry Biomass			
		Roots (kg ha^−1^)	Stems (kg ha^−1^)	Leaves (kg ha^−1^)	Total (kg ha^−1^)
CK	97.6 ± 3.9 a	303.2 ± 15.6 a	221.3 ± 12.2 a	470.0 ± 30.9 a	1004 ± 14 a
LDHs	98.9 ± 1.0 a	305.2 ± 35.7 a	234.0 ± 12.4 a	458.4 ± 22.9 a	998 ± 7 a

Note: Values are means ± SD (*n* = 3). Different letters indicate significant differences between treatments (*p* < 0.05).

**Table 2:** Cd mass-balance during moxa combustion under CK and LDH treatments.

Treatments	Leaves (µg g^−1^)	Moxa (µg g^−1^)	Ash (µg g^−1^moxa)	Smoke (µg g^−1^moxa)
CK	35.5 ± 5.8 a	34.4 ± 4.6 a	20.4 ± 4.0 a	14.1 ± 0.9 a
LDHs	23.5 ± 4.5 b	23.4 ± 1.1 b	14.0 ± 1.8 b	9.4 ± 1.9 b

Values are means ± SD (*n* = 3). Different lowercase letters within the same column indicate significant differences between CK and LDH treatments at *p* < 0.05. The mass of Cd per unit of moxa combusted is shown for initial moxa and residual ash. Cd released into smoke was estimated by mass-balance as the difference between Cd in moxa and Cd retained in ash (ΔCd_smoke_ = Cd_moxa_ − Cd_ash_).

## Data Availability

The original contributions presented in this study are included in this article. Further inquiries can be directed to the corresponding authors.
